# Habitat environmental factors influence intestinal microbial diversity of the short-faced moles (*Scaptochirus moschata*)

**DOI:** 10.1186/s13568-021-01252-2

**Published:** 2021-06-23

**Authors:** Lei Chen, Di Xu, Jing Zhu, Shen Wang, Mi Liu, Mengyao Sun, Geyang Wang, Lingyu Song, Xiaoyu Liu, Tianyu Xie

**Affiliations:** College of Life Science, Qufu Normal University, Jingxuan West Street No.57, Qufu, Shandong Province 273165 China

**Keywords:** Short-faced moles, Gut microbiome, Gender, Sampling locations, Edaphic factor

## Abstract

The short-faced moles (*Scaptochirus moschata*) are unique Chinese mammal that live in burrows for life. They have complex ecological adaptation mechanisms to adapt to perennial underground life. Intestinal microbes play an important role in the ecological adaptation of wild animals. The gut microbiota diversity and its function in short-faced moles’ ecological adaptation is a scientific issue worth exploring. In this study, the Illumina HiSeq sequencing platform was used to sequence the V3-V4 hypervariable regions of the 16S rRNA genes of 22 short-faced moles’ intestinal samples to study the composition and functional structure of their intestinal microbiota. The results showed that in the short-faced moles’ intestine, there are four main phyla, *Firmicutes, Proteobacteria, Actinobacteria* and *Bacteroidete*. At the family level, *Peptostreptococcaceae* and *Enterobacteriaceae* have the highest abundance. At the genus level, *Romboutsia* is the genus with the highest microbial abundance. According to the KEGG database, the main functions of short-faced mole gut microbes are metabolism, genetic information processing, environmental information processing, and cellular processes. The function of short-faced mole intestinal microbiota is suitable for its long-term burrowing life. No gender difference is found in the composition and function of the short-faced mole intestinal microbiota. There are significant differences in the composition and functional structure of the short-faced mole gut microbiota between samples collected from different habitats. We conferred that this is related to the different environment factors in which they live, especially to the edaphic factors.

**Supplementary Information:**

The online version contains supplementary material available at 10.1186/s13568-021-01252-2.

## Introduction

Short-faced mole (*Scaptochirus moschata*) belongs to the Mogera of the Talpidae, is a small mammal that lives in burrows for life, feeds on plant rhizomes and soil insects such as grubs, etc. It has developed morphological and physiological characteristics adapted to burrowing, such as eye degeneration, foreclaw valgus, smell and hearing sensitivity, etc. Short-faced mole is Chinese endemic species, mainly distributed in Henan, Shandong, Inner Mongolia, Ningxia, Shanxi and other provinces in China. Several studies on Talpidae have focused on the phylogenetic and morphological evolution, while the ecological adaptability of Talpidae is less studied.

Intestinal microbes are important symbionts in mammalian gut and occupy important niche (Willey et al. 2014; Saxena and Sharma 2016). They play an important role in maintaining intestinal health and immune function, including in the proliferation of intestinal cells, defense against pathogens, metabolism of secondary products, and digestion of complex compounds (Flint et al. 2012). Intestinal microbes can also affect animal nutrition metabolism and intestinal immunity through intestinal-brain axis (Schoster et al. 2017; Li et al. 2018; Wang et al. 2007), which is of great significance to animal survival and environmental adaptation (Wu et al. 2016; Zhu et al. 2011; Chen et al. 2017). There are many factors that affect gut microbes, including the genetic background of the host, the diet, age, gender, social class of the host, and the climate of the habitat. Soil, as an essential part of terrestrial mammal habitat, also plays an important role in the composition and diversity of animal gut microbes (Zhao et al. 2018; Wang et al. 2019).

Short-faced moles live in an underground hypoxic environment all year round. The physiological adaptation mechanisms are important and representative in the ecological research of burrowing mammals. The functional contribution of intestinal microbes in the burrowing life of the short-faced moles is an important field in the study of the physiological ecology of the burrowing mammals. Therefore, this study intends to study the gut microbial diversity of the short-faced moles, and to investigate the differences of intestinal microbes in moles with different sex and distribution. Furthermore, through the prediction of microbiome function, we also try to explore the function of gut microbes and their possible contribution in the adaptation to underground life of the short-faced moles. This study will provide data for the understanding of the physiological and ecological mechanism of short-faced moles to adapt to burrowing life, and provide data for the ecological studies of small burrowing mammals.

## Materials and methods

### Samples collection

In this study, 22 short-faced moles (SM) were collected using non-damagingly self-made mouse traps in September 2020, including 12 males (named M) and 10 females (named F). The sampling locations were Guanxian (115°E, 36°N; named G; 11) and Huimin (117°E, 37°N; named H; 11). The short-faced moles were captured and placed in the soil of the original habitat, and transported to the laboratory alive. Subsequently, the gasified diethyl ether was put into the transparent sealing device, and then the collected samples were put into the device, and the diethyl ether was continued. During this process, the state of short-faced moles was kept observing. After euthanasia, short-faced moles were dissected and the colon contents were collected aseptically. The collected colon contents samples were stored at – 80℃ in the laboratory until DNA extraction.

This study was approved by the Medical Ethics Committee of Qufu Normal University (No. 2020018), and during the approval period, the short-faced moles was captured and dissected as required.

### DNA extraction, PCR amplification and sequencing

We used QIAamp DNA Stool Genomic DNA Extraction Kit (Qiagen, Germany) to extract DNA from intestinal samples according to the instructions. The concentration of the DNA was detected by NanoDrop™ OneC (Thermo Fisher Scientifc, USA). We used qualified DNA samples for PCR amplification, then diluted the sample to 1 ng/μl using sterile water. PCR amplification of the V3-V4 region of 16S rRNA gene was performed with the primers 341F (CCTAYGGGRBGCASCAG) and 806R (GGACTACNNGGGTATCTAAT). The PCR reaction steps were as follows: pre-denaturation at 98 °C for 1 min; denaturation at 98 °C for 10 s, annealing at 55 °C for 30 s, extension at 72 °C for 30 s (35 cycles); and final extension at 72 °C for 5 min.

The concentration of PCR amplified products was detected by 2% agarose gel electrophoresis. Then the gel extraction kit (Qiagen, Germany) was used to purify the PCR products. The TruSeq® DNA PCR-Free Sample Preparation Kit was used to construct the library. The constructed library was quantitatively tested by Qubit. After the library was qualified, the Illumina HiSeq sequencing platform was used for sequencing.

### Sequencing data preprocessing

According to the quality control process of Qiime (V1.9.1) (Caporaso et al. 2010), the raw tags obtained by sequencing were filtered strictly by quality control to obtain high-quality clean tags. Truncate Raw Tags from the first low-quality base site where the number of consecutive low-quality values (the default quality threshold is <  = 19) has reached the set length (the default length is 3). Then filter out the Tags whose continuous high-quality base length is less than 75% of the Tag length. After detect and remove chimeric sequences, the final effective tags were obtained for further analysis.

### OTUs classification and species abundance analysis

To detect the species richness in each sample, the effective tags in all samples is classified as operational taxonomic units (OTUs) based on 97% sequence similarity. Then the OTUs were compared to SILVA's SSUrRNA database for species annotation (set threshold of 0.8) by using Mothur software (Version 1.35.1). R software (v2.15.3) was used to generate Rarefaction curve and Rank Abundance curve. The end of the Rarefaction curve is gradually flattened, which proves the rationality of the sequencing amount, and the results obtained by sequencing can effectively reflect the abundance of microorganisms contained from the sample. We analyzed the OTUs distribution to study the species abundance of the gut microbes of each sample, and constructed a histogram of the relative abundance of OTUs at each classification levels (phylum, class, order, family, and genus).

### Diversity analysis and significant test

To analyze the diversity of the microbial community intragroup, we performed alpha diversity analysis by using Mothur software (Version 1.35.1) to reflect the abundance and diversity of the microbial community of each sample, including the observed-species (OTUs) abundance, chao1, Shannon, Simpson, and ACE indices (Li et al. 2013). Wilcoxon rank sum test was performed by using R software to evaluate the difference among the alpha diversity indices of different groups. R software was also used for beta diversity analysis based on Unweighted & Weighted Unifrac distances to compare the microbial community composition intergroup. Multivariate statistical methods, include Principal Component Analysis (PCA), Principal Co-ordinates Analysis (PCoA) and Non-Metric Multi-Dimensional Scaling (NMDS) were performed to find the differences between different sample groups (Lozupone et al. 2005; Lozupone et al. 2011).

We used R vegan software package to perform Bray–Curtis distance-based Anosim (Chapman et al. 1999), MRPP (O'Reilly et al. 1980) and ADONIS (Stat et al. 2013) analysis to assess whether the difference between groups was significantly higher than the difference within groups. Mothur software was used for AMOVA (Roewer et al. 1996) based on weighted and unweighted Unifrac distance to compare microbial diversity between different groups. LEfSe (Segata et al. 2011) was used to analyze the difference of abundance on each classification level (phyla, class, order, family, and genus) between groups, and to find the biomarkers that have a significant contribution to the difference between groups. Unweighted Pair-group Method with Arithmetic Means (UPGMA) trees based on Unweighted & Weighted Unifrac distance were constructed to assess the similarity of the intestinal microbial diversity between different samples.

### Function prediction of short-faced moles intestinal microbiota

We perform Tax4Fun function prediction by the Neighbor-Joining test based on minimum 16S rRNA gene sequence similarity. We extracted the 16S rRNA gene sequence of the prokaryotic whole genome from the KEGG database and used the BLASTN algorithm to compare it to the SILVA SSU Ref NR database (BLAST bitscore > 1500) to establish a correlation matrix. The KEGG database prokaryotic genome function information annotated by UProC and PAUDA methods was corresponding to the SILVA database to realize the function annotation of the SILVA database. Sequencing samples were clustered with the SILVA database sequence as the reference sequence OTUs, thus the functional annotation information was obtained (Aßhauer et al. 2015).

## Results

### Sequencing data processing and OTU analysis

**Fig. 1:**
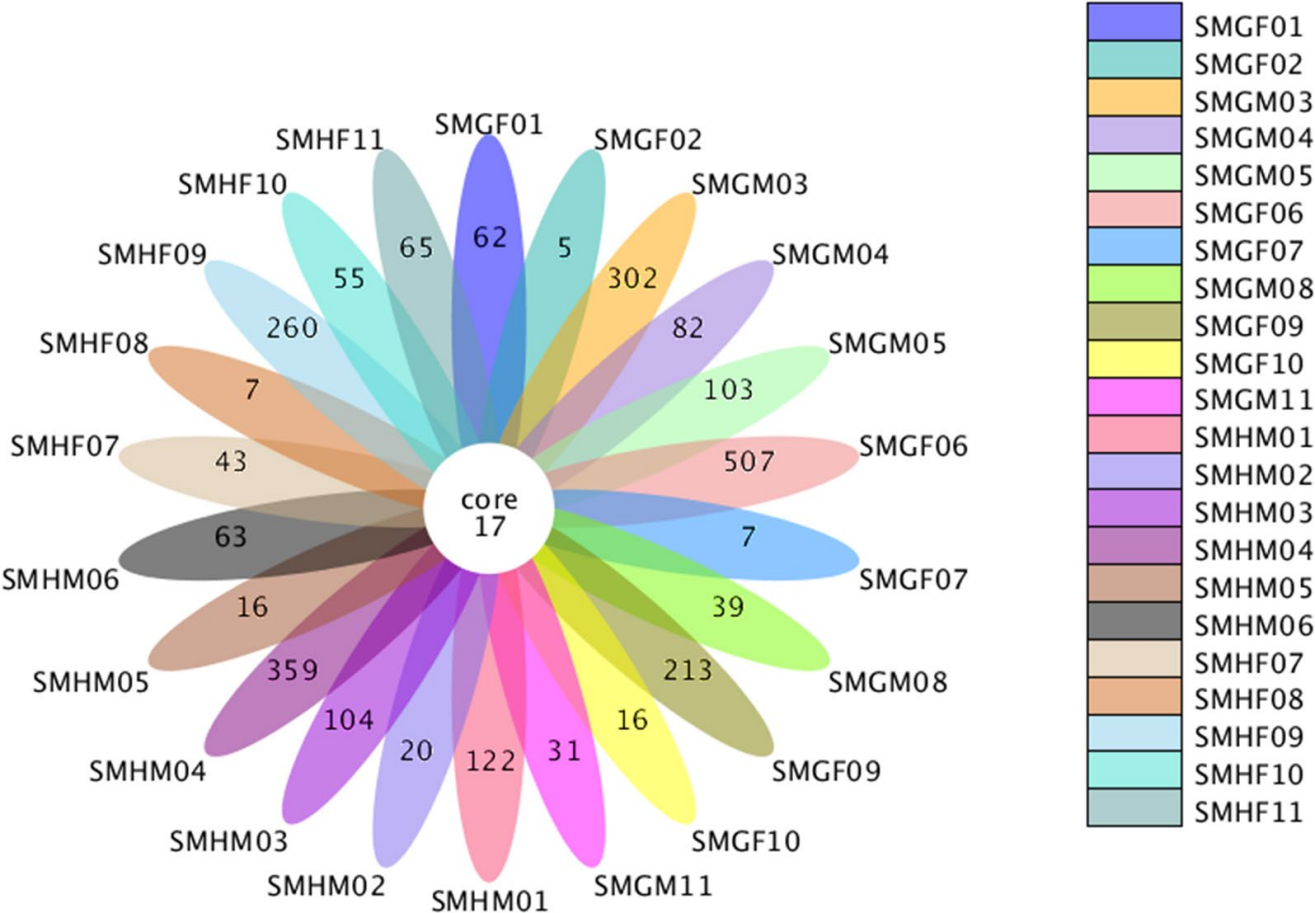
The core number represents the number of OTUs shared by all samples

A total of 2,085,117 raw paired-end reads are obtained from 22 short-faced moles intestinal samples. After quality control and chimera filtering, a total of 1,367,088 effective tags can be used for subsequent analysis with an average length of 411.91 bp. By clustered with 97% consistency, an average of 283 OTUs is obtained for each sample. According to the flower figure, there are only 17 OTUs shared by 22 samples, and the specificity between samples is relatively large (Fig. 1). The rarefaction curve and rank abundance curve indicate that sample size in this study is sufficient and can be used for subsequent analysis. Furthermore, the rarefaction curve (Additional file 1: Fig. S1) indicates that the intestinal microbial species abundance of SMGF06 is significantly higher than other samples. The rank abundance curve (Additional file 2: Fig. S2) also shows that the species abundance of SMGF06 samples is higher than that of other samples, but there is no obvious difference in species evenness.

### Gut microbiota composition of short-faced moles

Except for unclassified microbial sequences and a little percentage of archaea, the short-faced moles intestinal microbiota includes 46 bacterial phyla, 60 classes, 138 orders, 270 families and 688 genera.

**Fig. 2:**
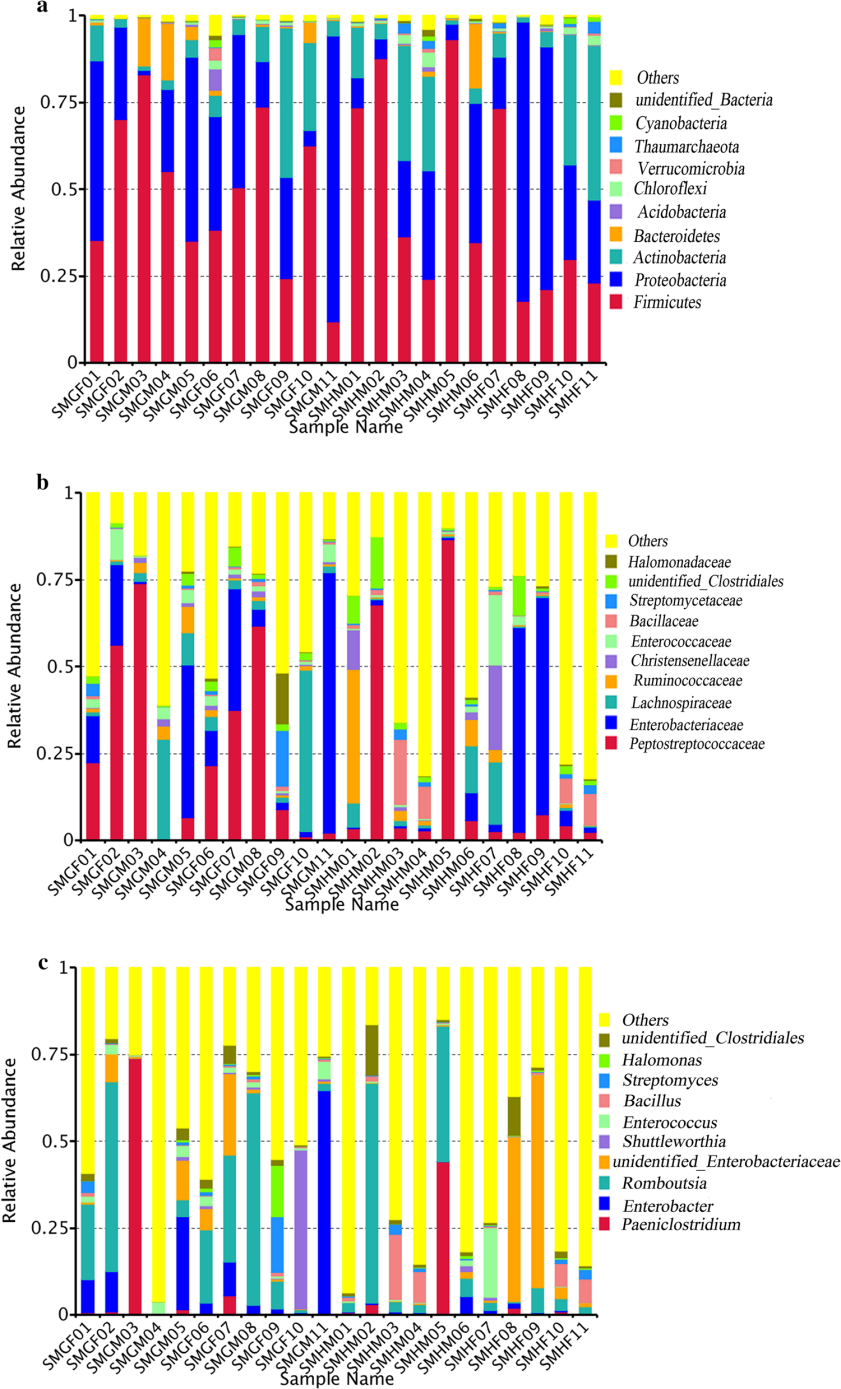
Column chart of relative abundance of species at phylum (**a**), family (**b**), and genus (**c**) level

At the phylum level, *Firmicutes* (47.91%) is the most abundant bacterial phylum, followed by *Proteobacteria* (31.36%), *Actinobacteria* (13.43%), *Bacteroidete* (3%), *Chloroflexi* (0.97%), *Thaumarchaeota* (0.55%), *Acidobacteri* (0.525%), *Cyanobacteria* (0.37%) and *Verrucomicrobia* (0.32%) (Fig. 2a). According to the sample collection area, we divided the samples into group Guanxian (SMG) and group Huimin (SMH). The bacterial phyla *Candidatus_Jorgensenbacteria, Acetothermia, Gracilibacteria, Parcubacteria, Candidatus_Yanofskybacteria, Atribacteria, Candidatus_Nomurabacteri, Zixibacteria* and *Candidatus_Moranbacteria* are only found in group SMG; phyla *Candidatus_Kuenenbacteria, Berkelbacteria, Candidatus_Peregrinibacteria, Kiritimatiellaeota, Lentisphaerae* and *Candidatus_Woykebacteria* are only found in group SMH (Additional file 3: Fig. S3). According to the gender of the samples, we divided the 22 samples into a male group (SMM) and a female group (SMF). The bacterial phyla of *Candidatus_Kuenenbacteria, Crenarchaeota, Acetothermia, Berkelbacteria, Candidatus_Peregrinibacteria, Kiritimatiellaeota, Lentisphaerae* and *Candidatus_Woykebacteria* are only found in group SMM; *Parcubacteria, Hydrogenedentes, Candidatus_Yanofskybacteria, Candidatus_Nomurabacteria, Zixibacteria, Candidatus_Moranbacteria* are only found in group SMF (Additional file 4: Fig. S4).

At the family level, the top ten abundant families are Peptostreptococcaceae (21.85%), Enterobacteriaceae (15.95%), Lachnospiraceae (6.63%), Ruminococcaceae (3.56%), Enterococcaceae (2.65%), Bacillaceae (2.52%), Christensenellaceae (2.30%), Micrococcaceae (1.72%), Streptomycetaceae (1.49%) and Microbacteriaceae (1.23%) (Fig. 2b). At the genus level, Romboutsia (15.27%) is the most abundant genus, followed by nidentified_Enterobacteriaceae (7.77%), Enterobacter (6.34%), Paeniclostridium (6.16%), Shuttleworthia (2.37%), Bacillus (2.27%), Enterococcus (2.16%), Streptomyces (1.49%), Halomonas (0.89%) (Fig. 2c).

### Comparison of intestinal microbial diversity between groups

To test the similarity of microbial diversity in different samples from the same sampling location, we performed Anosim analysis based on the Bray–Curtis distance. The result showed that SMH vs SMG R = 0.06965, P = 0.06, which proved that the difference between groups was greater than the difference within groups (Additional file 5: Fig. S5). The Anosim analysis of the groups SMF and SMM showed that R = 0.0009, P = 0.451, proving that the difference between groups is also greater than the difference within the group (Additional file 6: Fig. S6). MRPP analysis and AMOVA analysis were also performed to further verify this result. The MRPP results showed that SMG vs SMH A = 0.01714, P = 0.036, and SMF vs SMM A = 0.0004643, P = 0.405. The AMOVA analysis also showed that the difference both between the location groups and the gender groups was greater than that within the group, but the difference was all not significant (SMG vs SMH: weighted Unifrac AMOVA analysis, P = 0.396; unweighted, P = 0.118. SMF vs SMM: weighted Unifrac AMOVA analysis, P = 0.306; unweighted, P = 0.31).

We performed multiple regression analysis to explore whether there were significant differences in the gut microbes of short-faced moles between genders and between sample sites. The results showed that there was no significant difference between the groups SMH and SMG, and between the groups SMF and SMM (Table 1).

**Table 1:** Multiple regression analysis of the influence of gender and sampling site on the alpha diversity of short-faced moles’ intestinal microbial diversity

Alpha index	OTUs	Shannon	Simpson	Chao1	ACE	Goods coverage	PD whole tree
Mean of SMF	1103	5.140	0.846	1326.504	1380.497	0.993	114.570
Mean of SMM	1046	5.053	0.795	1326.272	1341.789	0.993	93.808
Mean of SMH	1155	5.617	0.850	1437.842	1447.486	0.993	102.052
Mean of SMG	993	4.576	0.791	1214.934	1274.800	0.993	106.326
P value (SMF vs SMM)	0.725	0.826	0.428	0.932	0.813	0.622	0.389
P value (SMH vs SMG)	0.413	0.214	0.379	0.356	0.447	0.805	0.919

**Fig. 3:**
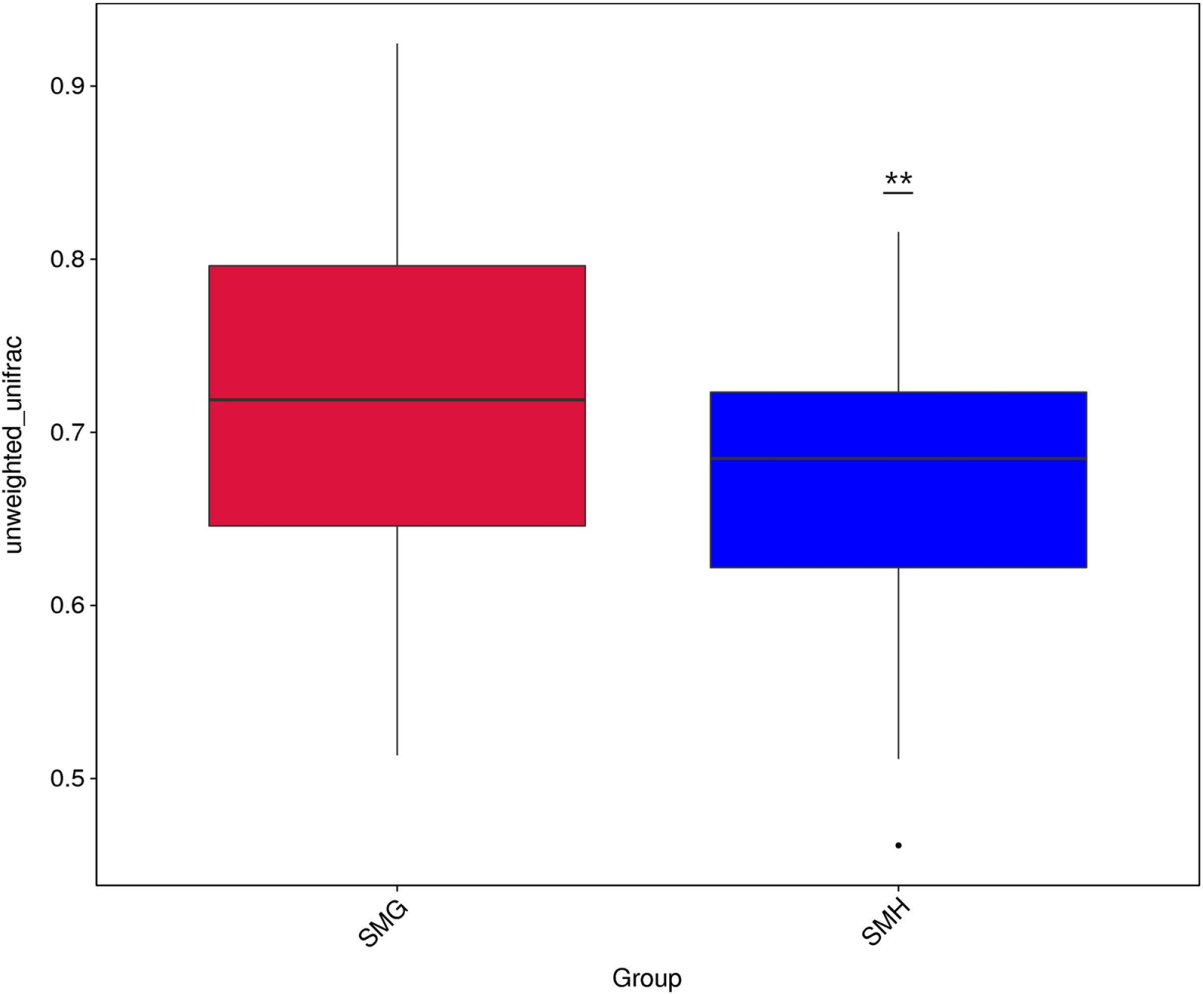
Beta diversity test between different sampling locations based on unweighted unifrac distance

Based on the weighted and unweighted Unifrac distance, we conducted a beta diversity test between different sampling locations. Beta analysis based on weighted Unifrac distance showed no significant difference between the groups SMG and SMH (P = 0.51), while beta diversity test based on the unweighted Unifrac distance showed extremely significant differences (P = 0.0033) (Fig. 3). Similarly, we also conducted a beta diversity test on the gut microbiota of different genders. The results showed that there was no significant difference between the groups SMM and SMF (weighted Unifrac beta analysis, P = 0.7346; unweighted Unifrac beta analysis, P = 0.4176). In addition, by PCoA, PCA and NMDS analysis, we further analyzed the similarity between different samples. However, results of PCoA, PCA and NMDS analysis show that there is neither sampling-related aggregation nor gender-related aggregation (Additional file 7: Fig. S7, Additional file 8: Fig. S8, Additional file 9: Fig. S9, Additional file 10: Fig. S10, Additional file 11: Fig. S11 and Additional file 12: Fig. S12).

**Fig. 4:**
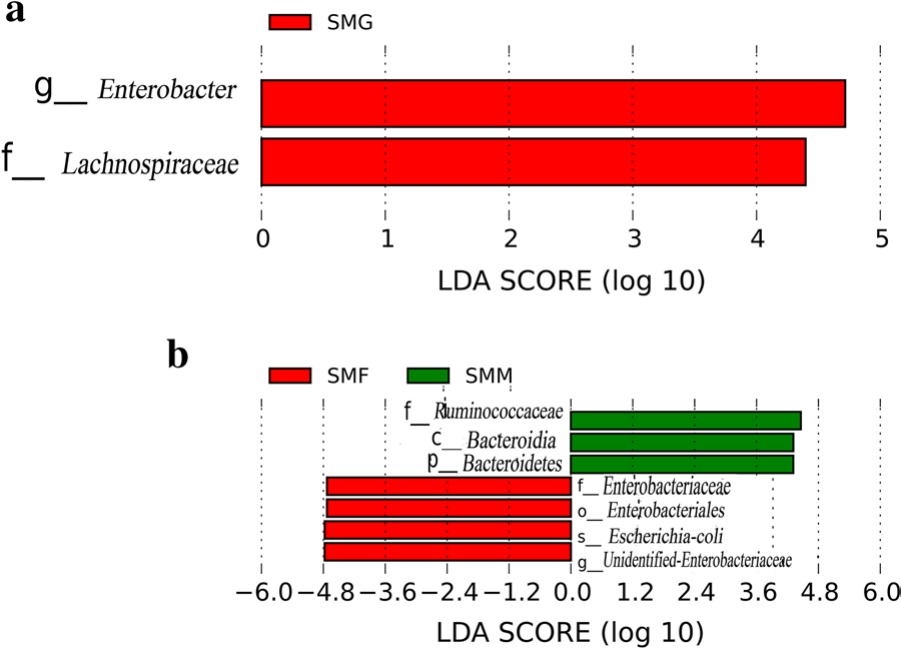
SMG-SMH **a** and SMF-SMM **b** LDA value distribution histogram

To further find out the differences between groups, we conducted wilcoxon tests between the groups on OTU abundance at each classification level (phylum, class, order, family, genus). There are a total of 6 phyla, 1 class, 2 orders, 5 families, and 23 genera have significant differences between groups SMG and SMH, including the phyla *Thaumarchaeota, Candidatus_Kuenenbacteria, Candidatus_Jorgensenbacteria, Berkelbacteria*; the class *Negativicutes*; the orders *Selenomonadales* and *Tepidisphaerale*; the families *Actinomycetaceae, Beutenbergiaceae, Paludibacteraceae, Actinomarinaceae, Parachlamydiaceae*; and the genera *Oxalobacter*, *Candidatus_Nitrososphaera*, *Rosenbergiella*, *Moryella*, etc. There are 5 phyla, 2 classes, 1 order, 4 families, and 23 genera with significant differences between groups SMM and SMF, including the phyla *Tenericutes, Candidatus_Kuenenbacteria, Crenarchaeota, Berkelbacteria, Acetothermia*; the classes *Thermoplasmata* and *Endomicrobia*; the order *Endomicrobiales*; the families *Paludibacteraceae, Endomicrobiaceae*, *Chromobacteriaceae, Parvularculaceae;* and the genera *Oxalobacter*, *Saccharofermentans*, *Moryella*, *Candidatus_ Nitrosotalea* and *Pirellula*, etc. Then LEfSe analysis was performed to find the biomarkers that contribute most to the significant differences between the two groups. Results of LEfSe analysis shows that the biomarker that have significant differences between SMG group and SMH group are family *Lachnospiraceae* (SMG 9.25%; SMH 4.01%) and genus *Enterobacter* (SMG 11.76%; SMH 0.91%) (SMG > SMH) (Fig. 4a). The biomarker between group SMM and group SMF are phylum *Bacteroidetes*; class *Bacteroidia*; family *Ruminococcaceae* (SMF 1.04%; SMM 6.09%) (SMF < SMM); order *Enterobacteriales*, family *Enterobacteriaceae* (SMF 19.50%; SMM 12.40%); genus *Unidentified-Enterobacteriaceae* and *Escherichia-coli* (SMF > SMM) (Fig. 4b).

### Prediction of intestinal microbial function

**Fig. 5:**
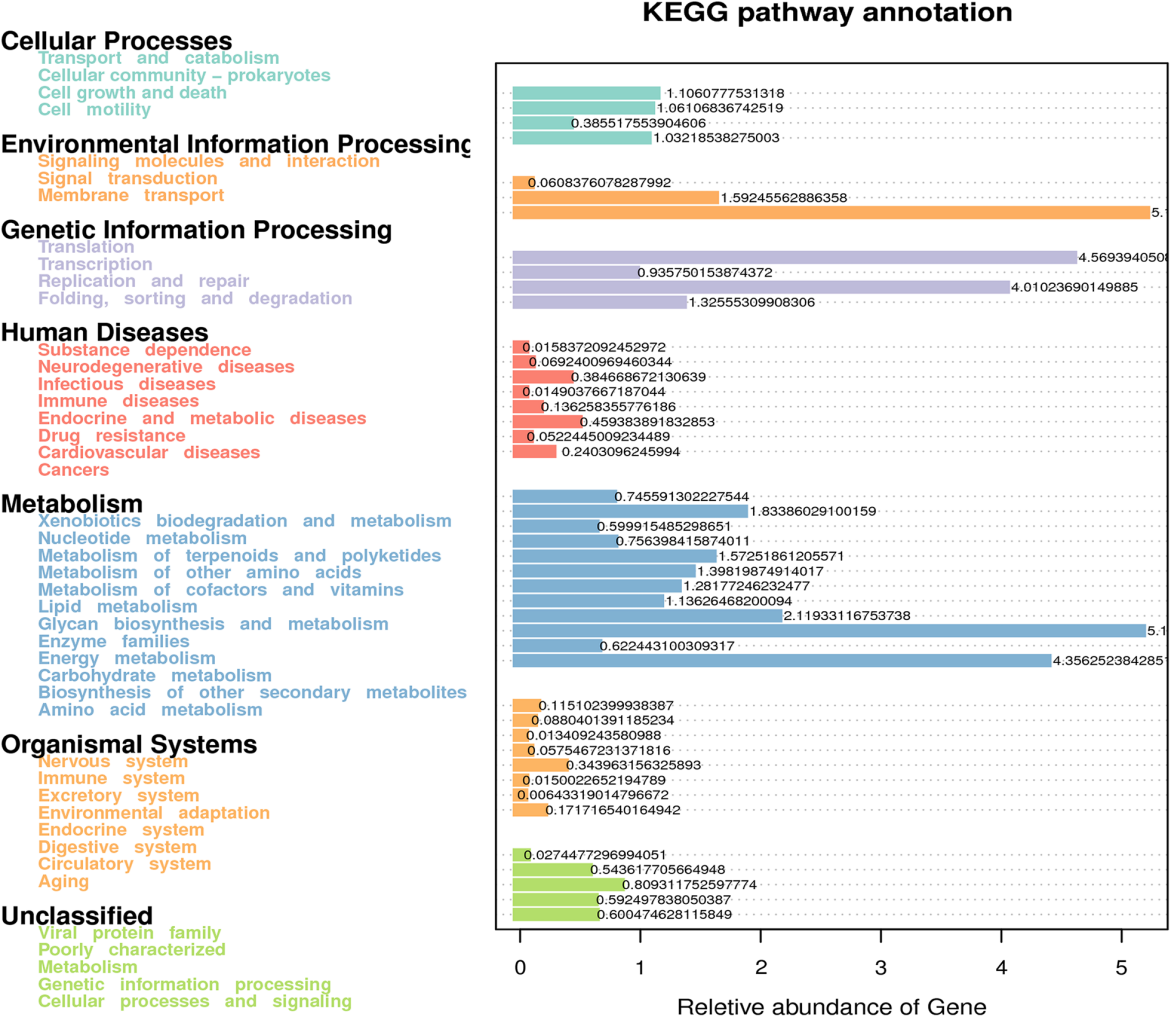
KEGG pathway annotation of short-faced moles gut microbiota

**Fig. 6:**
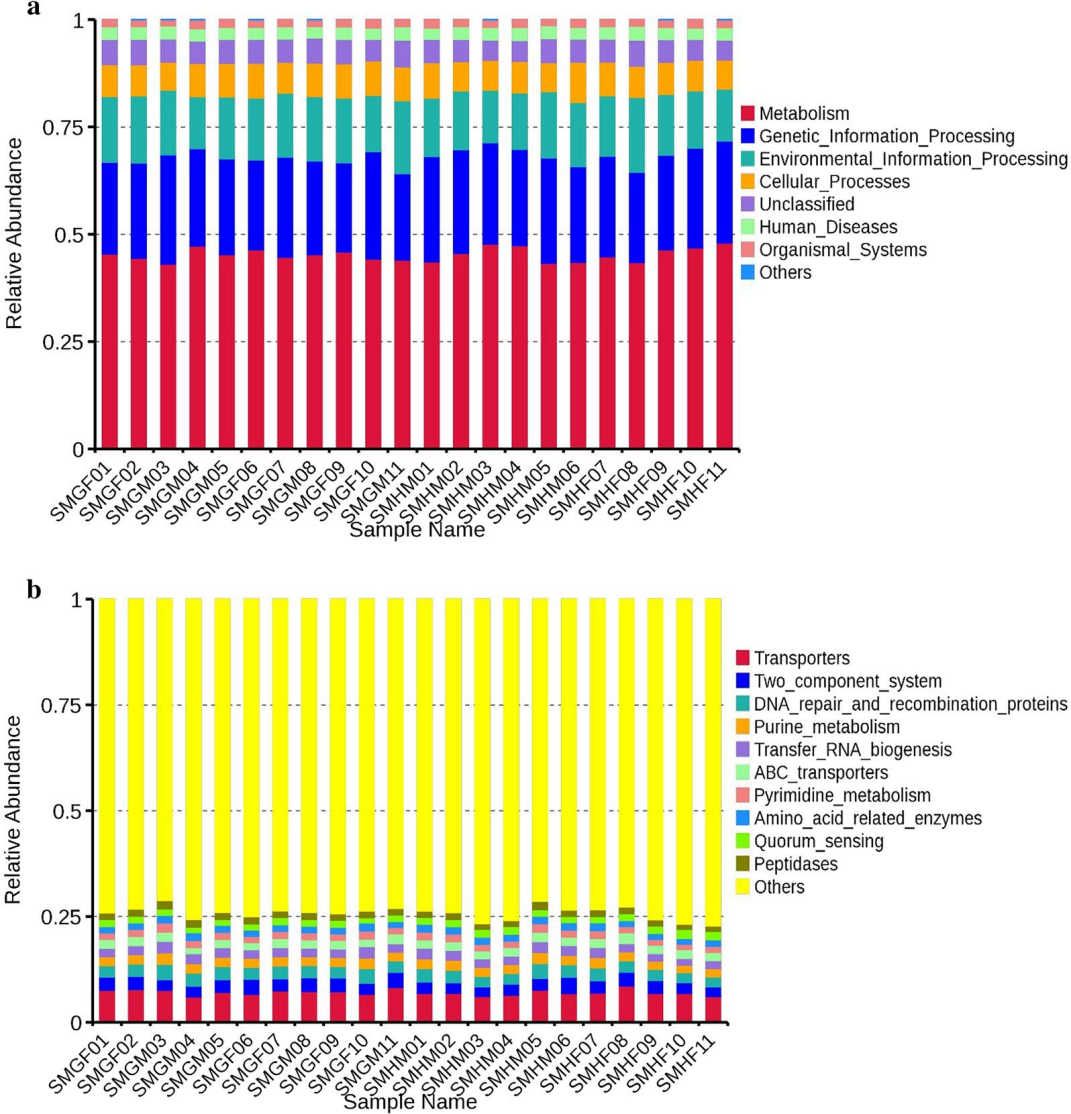
Tax4Fun Function annotation histogram of relative abundance of level 1 (**a**) and level 3 (**b**)

According to the function annotation information, the results show that the most important function of the intestinal microbiota of short-faced moles is metabolism (~ 45.30%), including carbohydrate metabolism (~ 10.81%), amino acid metabolism (~ 9.14%), energy metabolism (~ 4.45%), nucleotide metabolism (~ 3.86%), metabolism of cofactors and vitamins (~ 3.30%), lipid metabolism (~ 2.93%), etc. There is significant difference in the function abundance of carbohydrate metabolism and enzyme families between groups SMG and SMH by Wilcoxon test (P < 0.05). The second most important function is the genetic information processing (~ 21.38%), including translation (~ 9.59%), replication and repair (~ 8.44%), etc. There is significant difference in the function abundance of translation between the groups SMG and SMH (P < 0.01). The other important function is environmental information processing (~ 14.38%), including membrane transport (~ 10.90%), signal transduction (~ 3.35%), etc. The function of intestinal microbes of short-faced moles is also associated with several diseases, such as infectious diseases, drug resistance and cancer, etc. Groups SMG and SMH have significant differences in the function process of cancer (P < 0.05) (Fig. 5). At KEGG pathway level 3, the function of short-faced moles’ intestinal microbiota is mainly annotated to transporters, two component system, DNA repair and recombination proteins, purine metabolism, transfer RNA biogenesis, ABC transporters, pyrimidine metabolism, amino acid related enzymes, quorum sensing, peptidases, etc. (Fig. 6, Additional file 13: Fig. S13).

## Discussion

Short-faced mole is a small mammal that lives in underground caves all year round. Report on most aspects of short-faced mole remains scarce. In this study, we attempt to discuss the influence of gender and distribution area on the intestinal microbial diversity of the short-faced moles, and to find out whether there are significant differences in the gut microbes of short-faced moles of different genders or in different distribution locations. Through high-throughput sequencing of 22 short-faced moles’ intestinal samples of different genders collected from two habitats, 46 phyla, 60 classes, 138 orders, 270 families and 688 genera are detected in the short-faced moles intestinal samples. At the phylum level, *Firmicutes*, *Proteobacteria*, *Actinobacteria*, and *Bacteroidete* are the dominant phyla. This is consistent with the results of other mammals (Xu et al. 2015; Zhang et al. 2010; Chen et al. 2016; Han et al. 2019; Ley et al. 2008). Among them, the phylum *Firmicutes* occupies 47.91% of the total intestinal microbiota, and its abundance fluctuates greatly between different samples (11.86%–93.15%). Some rodents, such as mice (*Mus)*, have about 80% *Firmicutes* in their intestinal microbiota, and rat (*Rattus norvegicu)* contain *Firmicutes* at about 78.77% (Li et al. 2017). Among the herbivorous animals, such as bharals (*Pseudois nayaur)* (Chi et al. 2019), Tibetan wild ass (*Equus kiang)* (Gao et al. 2019), *Firmicutes* is also the dominant phyla of their gut microbes. Short-faced moles mainly feed on plant rhizomes, insect larvae, etc. The high abundance of *Firmicutes* can help short-faced moles enhance the digestion and absorption of plant cellulose.

*Proteobacteria* occupies 31.36% (1.19%-82.22%) of short-faced moles intestinal microbiota. The abundance of this phylum also varies greatly among individuals (82.22% in sample SMGM11, but only 1.19% in sample SMGM03). Previous studies have shown that *Proteobacteria* is related to malnutrition of hosts suffering from metabolic or inflammatory diseases, and may be related to the dynamic establishment of the gastrointestinal microbiota of young animals (Moon et al. 2018). However, the samples we collected did not show gastrointestinal diseases. Whether *Proteobacteria* are related to short-faced moles’ intestinal immunity needs further research.

*Actinobacteria* and *Bacteroidete* are also dominant phyla in the short-faced moles intestinal microbiota. Relevant studies have shown that an increase in protein and fat in animal foods will increase *Actinobacteria* abundance in intestinal microbiota (Guan et al. 2016). And carnivorous animals have high abundance of *Actinobacteria* in their gut may be associated with high fat and high protein feeding habits. In the present study, there is a high abundance of *Actinobacteria* in short-faced moles intestinal microbiota (second to *Proteobacteria*). We speculate that the diet of the short-faced moles may have a higher proportion of protein foods. Microbes of *Bacteroidetes* can promote digestion. They are the main bacteria that digest dietary polysaccharides and break down proteins. *Bacteroidetes* can help the host degrade high molecular weight organic matter, help the host degrade plant cell wall compounds (including cellulose, pectin and xylan), and play an important role in maintaining host intestinal health (Wu et al. 2011, 2016; Chen et al. 2017; Becker et al. 2014; Salyers et al. 1988). Compared with other mammals, such as bactrian camel (*Camelus bactrianus)*(20%) (Wang et al. 2018a, b); rat (*Rattus norvegicu)* (9.12%) (Li et al. 2017), the content of *Bacteroidetes* in the short-faced moles’ intestinal microbiota is low (only 3.00%), which is similar to the results of the study on the intestinal microbes of grey geese (*Anser anser*) (Wang et al. 2018a, b). According to the researchers, a low *Bacteroidetes* abundance of grey geese is due to dietary reasons, or other gut microbiota perform biopolymer degradation functions (Wang et al. 2018a, b). Therefore, we speculate that the low content *Bacteroidetes* in short-faced moles’ intestinal microbiota may be related to their food composition. And we speculate that the dietary polysaccharides in the short-faced moles’ diet take only a small percentage.

The beta diversity test results based on unweighted Unifrac distance showed that there are extremely significant differences between groups from different sampling locations. However, the beta diversity test results based on the weighted Unifrac distance showed no significant difference between the two groups. Study on the intestinal microbial community of free-range and captive cheetahs (*Acinonyx jubatus)* found that based on the weighted Unifrac distance analysis results have higher resolution than the results based on the unweighted Unifrac distance. Then they indicated that the difference between captive and free-range cheetahs is attributed more to the different abundance of several microbes than to the existence of the unique OTUs (Wasimuddin et al. 2017). We consider that the differences between different regional groups may also be caused by the differences in the abundance of some OTUs.

Previous studies have found that the composition and diversity of Arctic sediment is similar to that of local mammalian intestinal microbial flora (Wang et al. 2019). Researchers also found that the living environment of baboons (*Papio)*, especially the geological history of soil and the content of exchangeable sodium, significantly affect the intestinal microbial flora (Grieneisen et al. 2019). In the present study, one of the sampling site (Huimin) is mainly a plain formed by sediment deposition of the Yellow River, and it is about 1,000 m away from the Yellow River. The other sampling site (Guanxian) is the alluvial plain of the Yellow River. Because of the diversion of the ancient Yellow River, it is more than 8000 m away from the Yellow River channel. Though examining the physical and chemical properties of the soil in the mole burrows of two sampling sites, we found that the amount of organic matter and cation exchange in soil of Huimin is lower than that in Guanxian. There are significant differences in cation exchange capacity (cmol/kg), exchangeable sodium (cmol (Na^+^) / kg) and organic matter (g/kg) (SMG > SMH) in the soil between Huimin and Guanxian (Additional file 14: Table S1). Short-faced moles live in underground caves for a life time and get food from the soil. According to our testing, there are some significant differences in the soil chemistry between the two regions (Additional file 14: Table S1). Therefore, we speculate that soil factors will have a certain impact on the intestinal microbes of short-faced moles, but its influence mechanism is still unclear and needs further study.

UPGMA analysis found that the gut microbiota did not cluster associated with sampling locations. There are still large differences in gut microbes between different individuals in the same sampling location, which may be related to the difference in the microenvironment between individuals. Therefore, environmental factors in the microenvironment may have a more significant impact on the gut microbes of short-faced moles, which have a long-term burrowing underground life with poor migration ability. In addition, due to the number of samples from the same sampling location is still small, whether the microenvironment has more influence on the diversity of intestinal microbiota or the distribution area has more influence on intestinal microbiota steel needs further study.

Many studies have reported that intestinal microbial diversity in humans and many wild mammals is related to gender. Studies on the intestinal microbes of dholes (*Cuon alpinus*) found that the abundance of *Bacteroidetes* in the gut microbes of female samples was higher than that of males (Wu et al. 2016). Studies on golden takin (*Budorcas taxicolor bedfordi*) also found that the gut microbial diversity was affected by gender (Chen et al. 2017). In the present study, the results showed that there was no significant difference in the diversity and abundance of intestinal microbial communities in different gender groups by alpha diversity and beta diversity analysis. However, when compared the abundance of short-faced moles intestinal microbial abundance of different gender groups at different classification levels, we found that there were significant differences between male and female groups in 5 phyla, 2 classes, 1 order, 4 families and 23 genera. By LEfSe analysis, we found the biomarkers of significant difference between group SMM and group SMF, included family *Ruminococcaceae* (SMF < SMM) and family *Enterobacteriaceae* (SMF > SMM).

In this study, we find that the main functions of the intestinal microbiome of short-faced moles are metabolism, genetic information processing and environmental information processing, etc. By metagenome sequencing and function annotation of three wild giant panda intestinal samples, researchers identified homologous sequences with genomic genes encoding cellulase, glucosidase, xylanase, and xylanase. They believe that giant panda intestinal microbes play an important role in the metabolism of lactic acid, amino acids, xenobiotics, nucleotides, polysaccharides, vitamins and lipids in giant pandas (Zhu et al. 2011). A study on the Siberian tiger (*Panthera tigris*) suggests that the intestinal microbial gene function of the Siberian tiger is mainly related to carbohydrate metabolism subsystem and protein metabolism, respectively. Compared with breast-fed Siberian tiger cubs, the metabolism, translation, replication and repair of carbohydrates, amino acid metabolism, membrane transport and cofactor and vitamin metabolism in the intestinal microbial community of goat breast-fed Siberian tiger cubs decreased (He et al. 2018). Study on the intestinal microbial function of other small mammals, such as shrews (*Tupaia belangeri*), it is also found that most of the gene function of intestinal microbes are related to metabolism, and are more involved in amino acid metabolism and carbohydrate metabolism. We find that sugar metabolism (10.81%), amino acid metabolism (9.14%), energy metabolism (4.45%) and nucleotide metabolism (3.86%) account for a large proportion of the main metabolic function of short-faced moles intestinal microbiota*.* We speculate that this may because that, on one hand, it is beneficial to enhance the digestion and absorption ability of plant cellulose, and on the other hand, it can provide sufficiently energy to adapt to the low oxygen and lightless living environment, and meet the nutritional and energy needs of reproduction.

At the second level of KEGG pathway, the annotated gene function of short-faced moles’ intestinal microbiota includes membrane transport, protein translation and other functions besides sugar metabolism, amino acid metabolism and other basic metabolisms. Some studies have shown that compared with captive cheetahs (*Acinonyx jubatus*), the intestinal flora functions of wild cheetahs mainly focus on environmental adaptation, membrane transport, immune system and translation. In order to adapt to environmental requirements such as food supply, competition, territory and family scope, wild cheetahs have enhanced these functions of intestinal flora. A study also found that immune-related genes are enriched in gut microbes in camels (*Camelus bactrianus*) about two years old (He et al. 2019). The gene function of short-faced moles intestinal microflora annotation shows that intestinal microbes also play an important role in the nutrient transport and metabolism of the short-faced moles, which may also be suitable for the life habits of long-term excavation.

In summary, the present study uses high-throughput sequencing technology to study the abundance, diversity and function of short-faced moles gut microbes, and the differences between gender groups and sampling location groups, which will provide data for the study of nutritional ecology of short-faced moles and other small burrowing mammals. In addition to sampling location and gender, other factors may also affect the diversity and function of mammalian gut microbiota. In later studies, the intestinal microbial diversity will be further associated with a variety of ecological factors, and the interaction between the diversity of intestinal microbes and environmental factors will be further studied. The sample size will be increased to draw more reliable conclusions. From the perspective of the mutual beneficial symbiosis of gut microbes and their hosts, it will open a new window to clarify the ecological adaptation mechanism of lifelong burrowing mammals.

## Supplementary Information

**Additional file 1:Figure S1.** The rarefaction curve.

**Additional file 2: Fig. S2.** Rank abundance curve.

**Additional file 3: Fig. S3.** Composition of gut microbes at the phylum level in different sampling sites.

**Additional file 4: Fig. S4.** Intestinal microbial composition of different genders at the phylum level.

**Additional file 5: Fig. S5.** Analysis of differences between Anosim groups of different sampling locations.

**Additional file 6: Fig. S6.** Analysis of differences between Anosim groups of different genders.

**Additional file 7: Fig. S7.**PCoA analysis between groups of different sampling locations.

**Additional file 8: Fig. S8.** PCoA analysis between groups of different genders.

**Additional file 9: Fig. S9.**PCA analysis between groups of different sampling locations

**Additional file 10: Fig. S10.**PCA analysis between groups of different genders.

**Additional file 11: Fig. S11.**NMDS analysis between groups of different sampling locations.

**Additional file 12: Fig. S12.**NMDS analysis between groups of different genders.

**Additional file 13: Fig. S13.** Tax4Fun Function annotation histogram of relative abundance of level 2.

**Additional file 14: Table S1.** Soil physical and chemical properties in two regions (Mean±SD).

## Data Availability

The raw data of the short-faced moles’ intestinal microbiota 16S rRNA genes could be found in the NCBI SRA database (PRJNA613028).
