# Correction to: Habitat environmental factors influence intestinal microbial diversity of the short-faced moles (*Scaptochirus moschatus*)

**DOI:** 10.1186/s13568-022-01373-2

**Published:** 2022-03-03

**Authors:** Lei Chen, Di Xu, Jing Zhu, Shen Wang, Mi Liu, Mengyao Sun, Geyang Wang, Lingyu Song, Xiaoyu Liu, Tianyu Xie

**Affiliations:** College of Life Science, Qufu Normal University, Jingxuan West Street, No.57, Qufu, 273165 Shandong Province China

## Correction to: AMB Expr 11:93 (2021) https://doi.org/10.1186/s13568-021-01252-2

Following publication of the original article (Chen et al. 2021), the authors would like to correct the Latin name of the species (short-faced mole) in this article.

The correct Latin name of short-faced mole is *Scaptochirus moschatus* instead of *Scaptochirus moschata*. This has been corrected in this erratum.
