# Supplementary figures and images for: Habitat environmental factors influence intestinal microbial diversity of the short-faced moles (Scaptochirus moschata)
Source: AMB Express. 2021 Jun 23;11:93. doi: 10.1186/s13568-021-01252-2 (PMC8222469; doi:10.1186/s13568-021-01252-2)

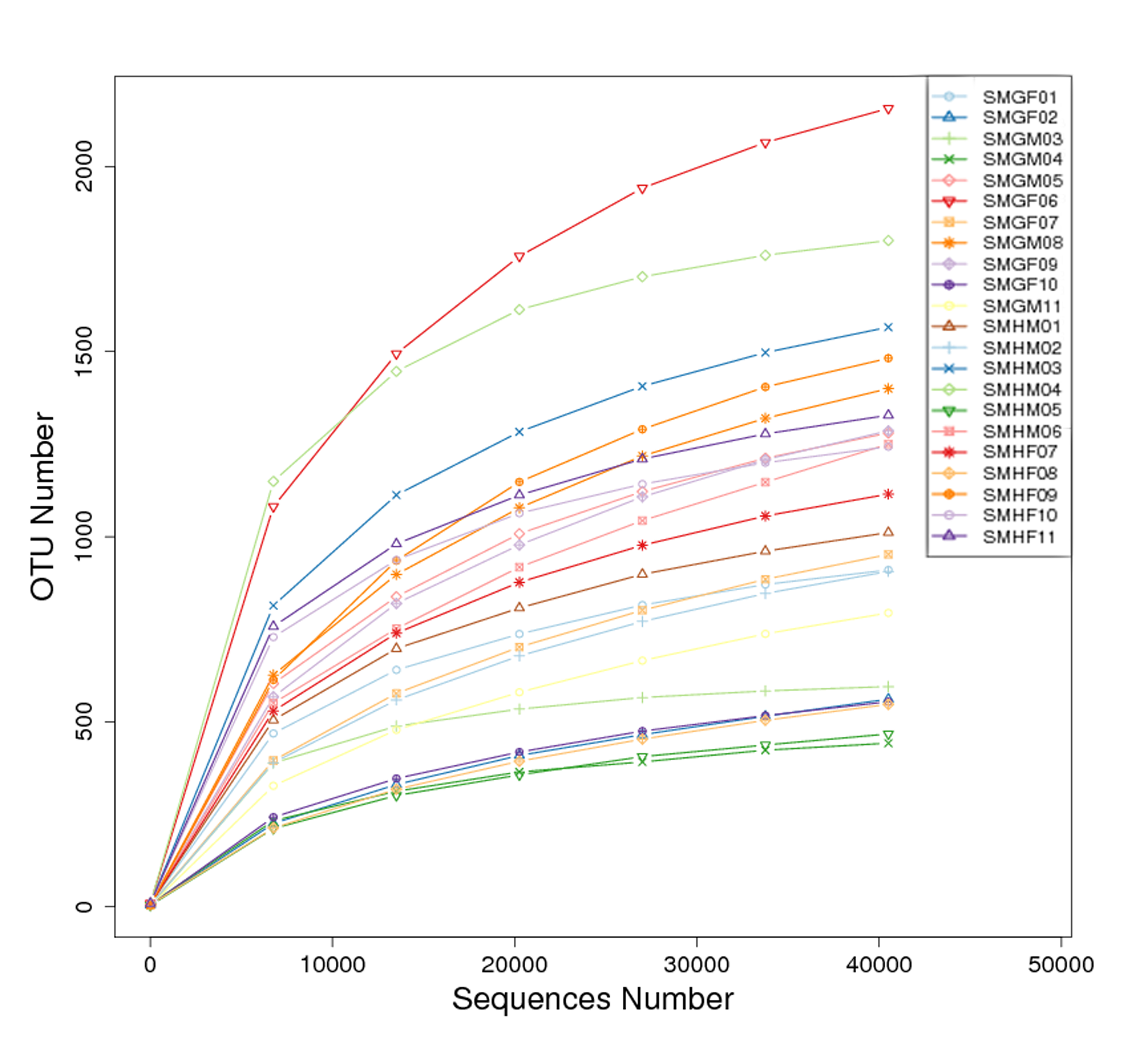

Supplement: Supplementary file 1 — Additional file 1:Figure S1. The rarefaction curve. [file 13568_2021_1252_MOESM1_ESM.jpg]

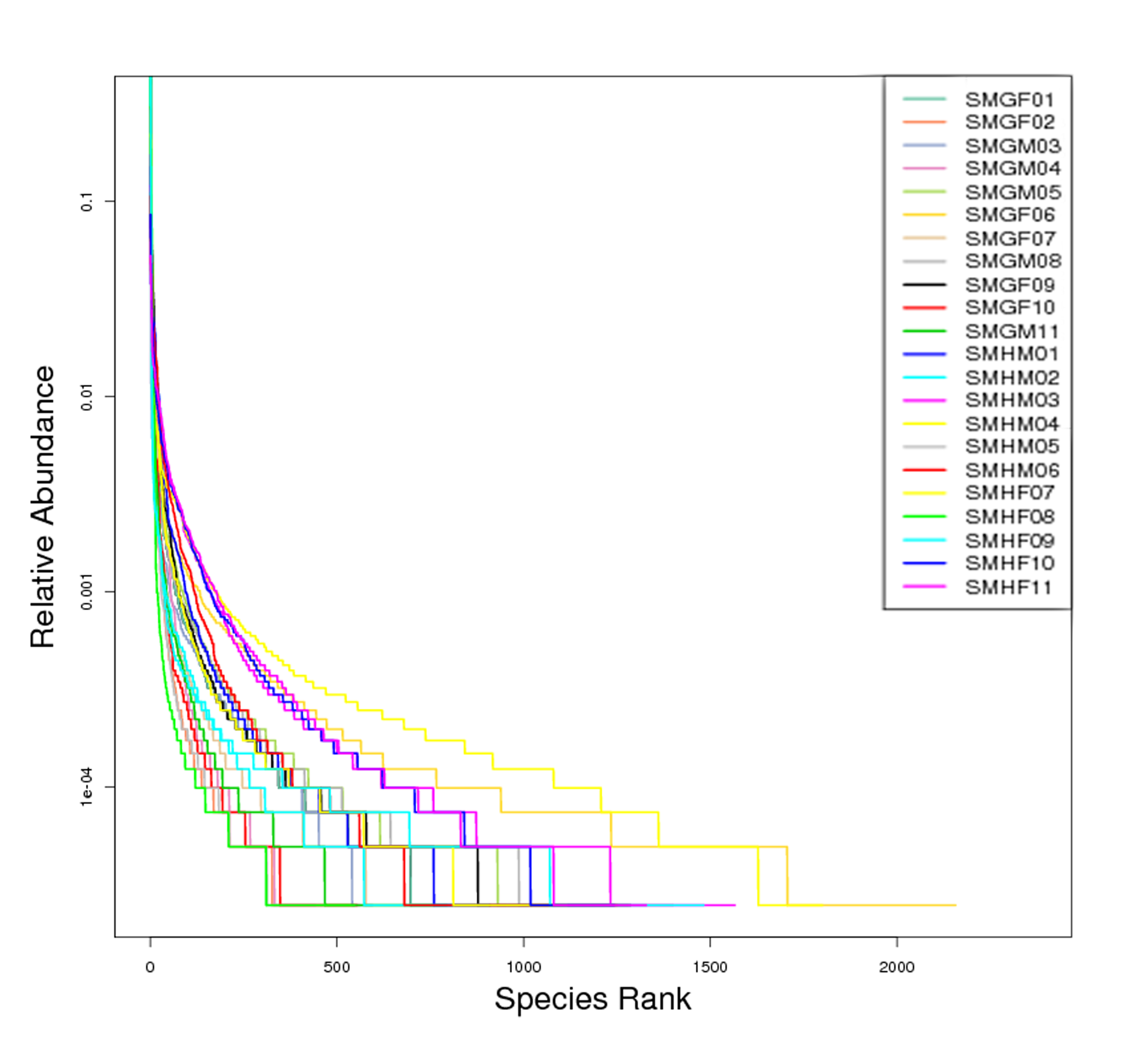

Supplement: Supplementary file 2 — Additional file 2: Fig. S2. Rank abundance curve. [file 13568_2021_1252_MOESM2_ESM.jpg]

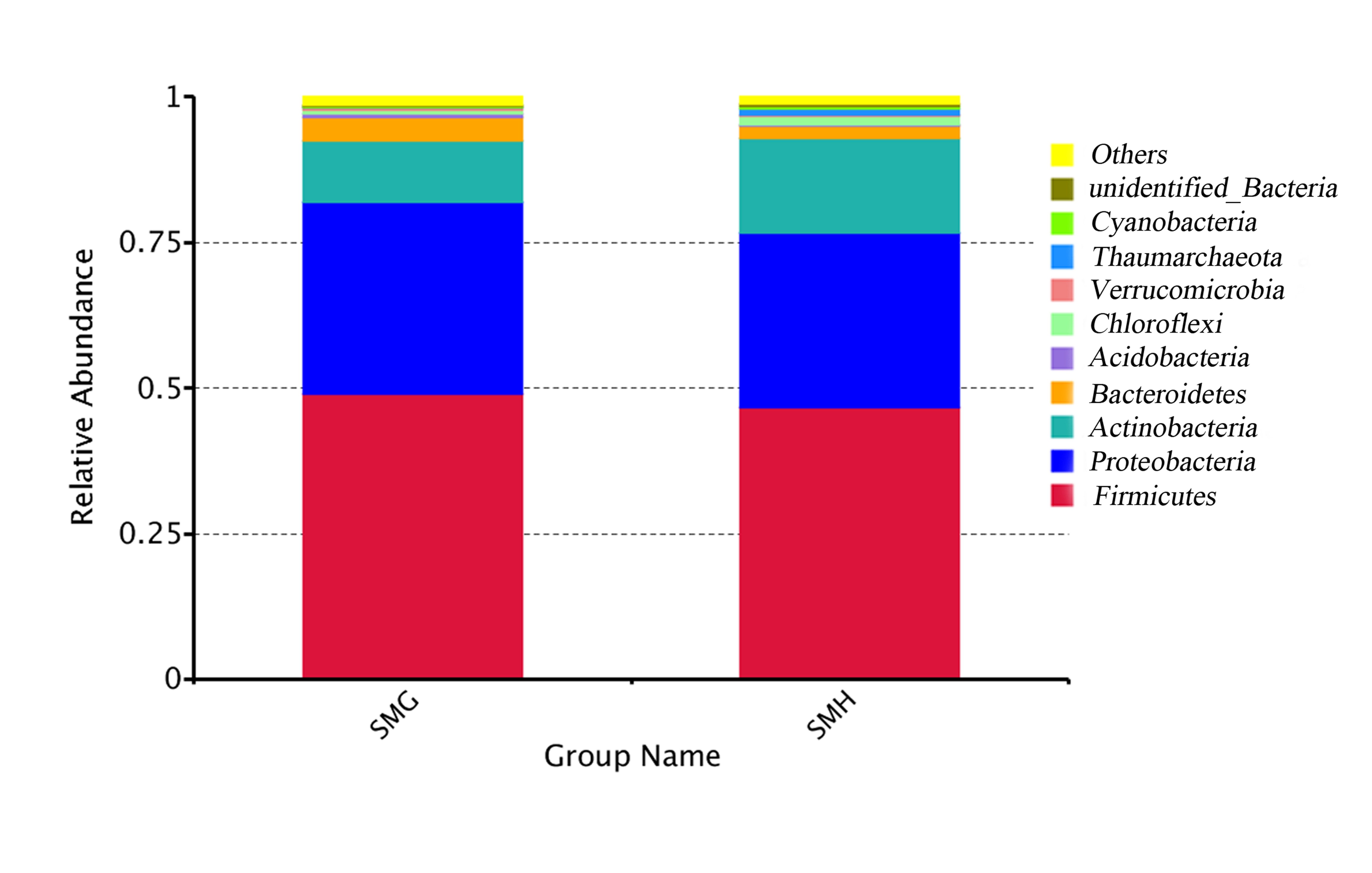

Supplement: Supplementary file 3 — Additional file 3: Fig. S3. Composition of gut microbes at the phylum level in different sampling sites. [file 13568_2021_1252_MOESM3_ESM.jpg]

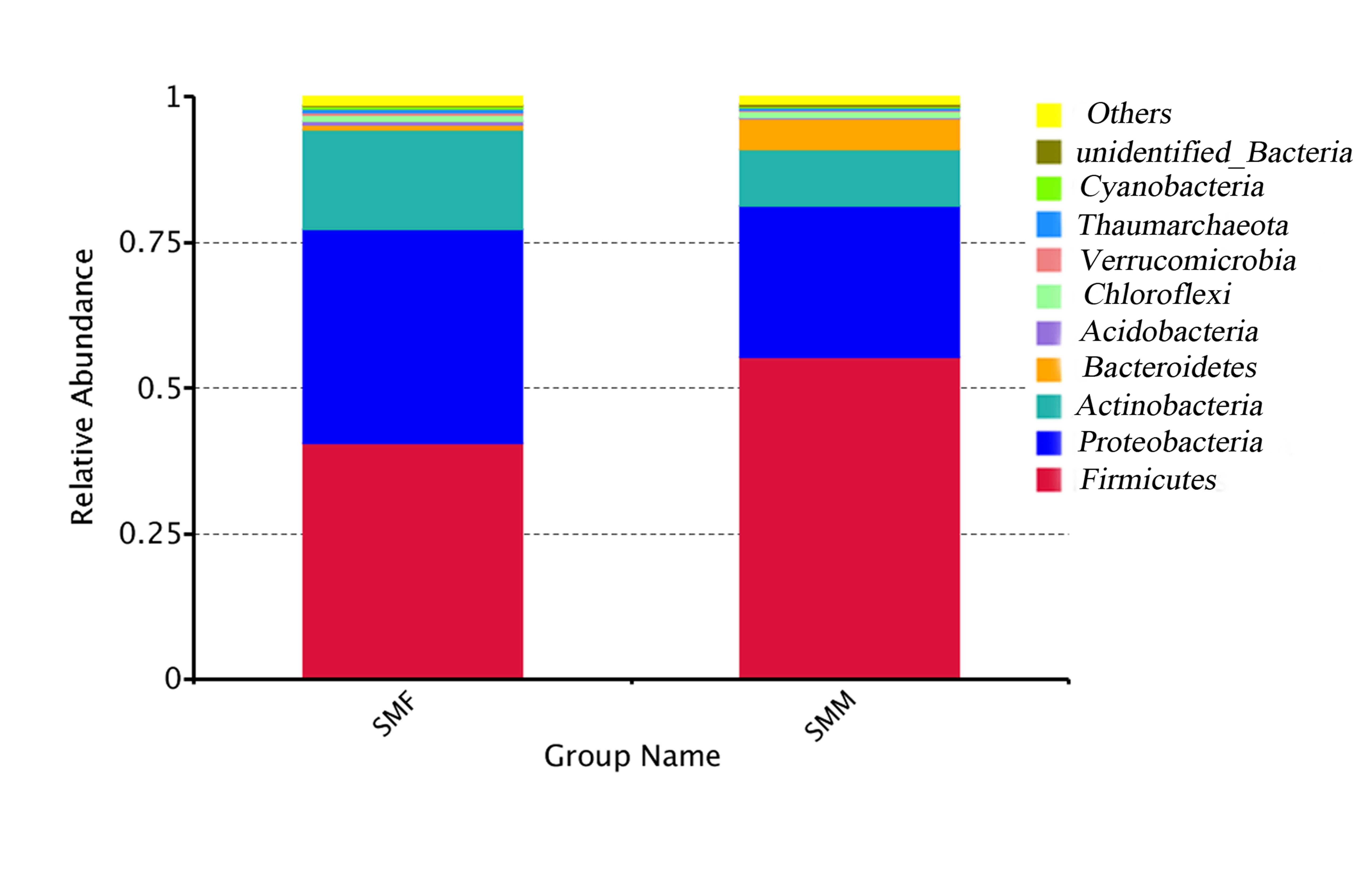

Supplement: Supplementary file 4 — Additional file 4: Fig. S4. Intestinal microbial composition of different genders at the phylum level. [file 13568_2021_1252_MOESM4_ESM.jpg]

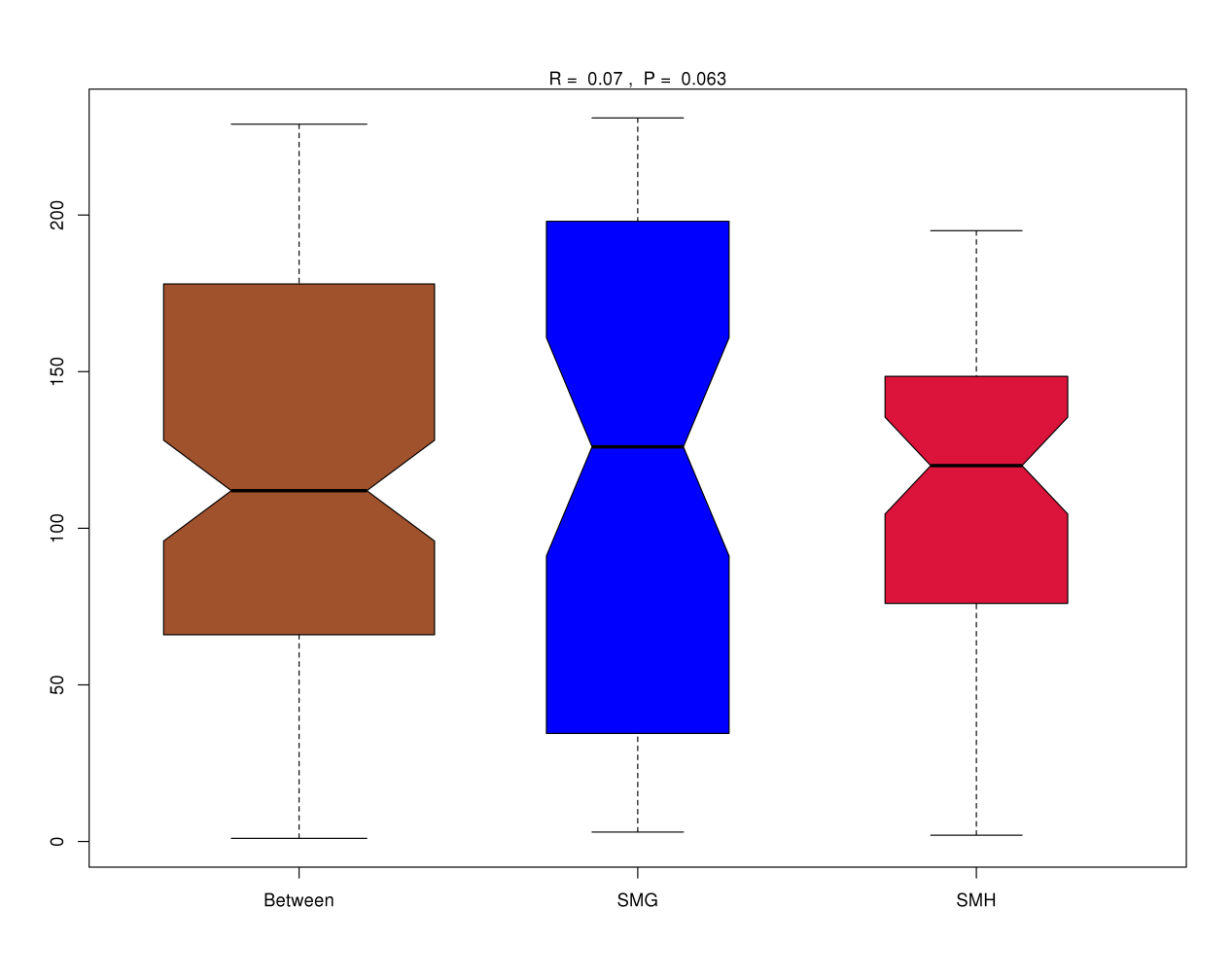

Supplement: Supplementary file 5 — Additional file 5: Fig. S5. Analysis of differences between Anosim groups of different sampling locations. [file 13568_2021_1252_MOESM5_ESM.png]

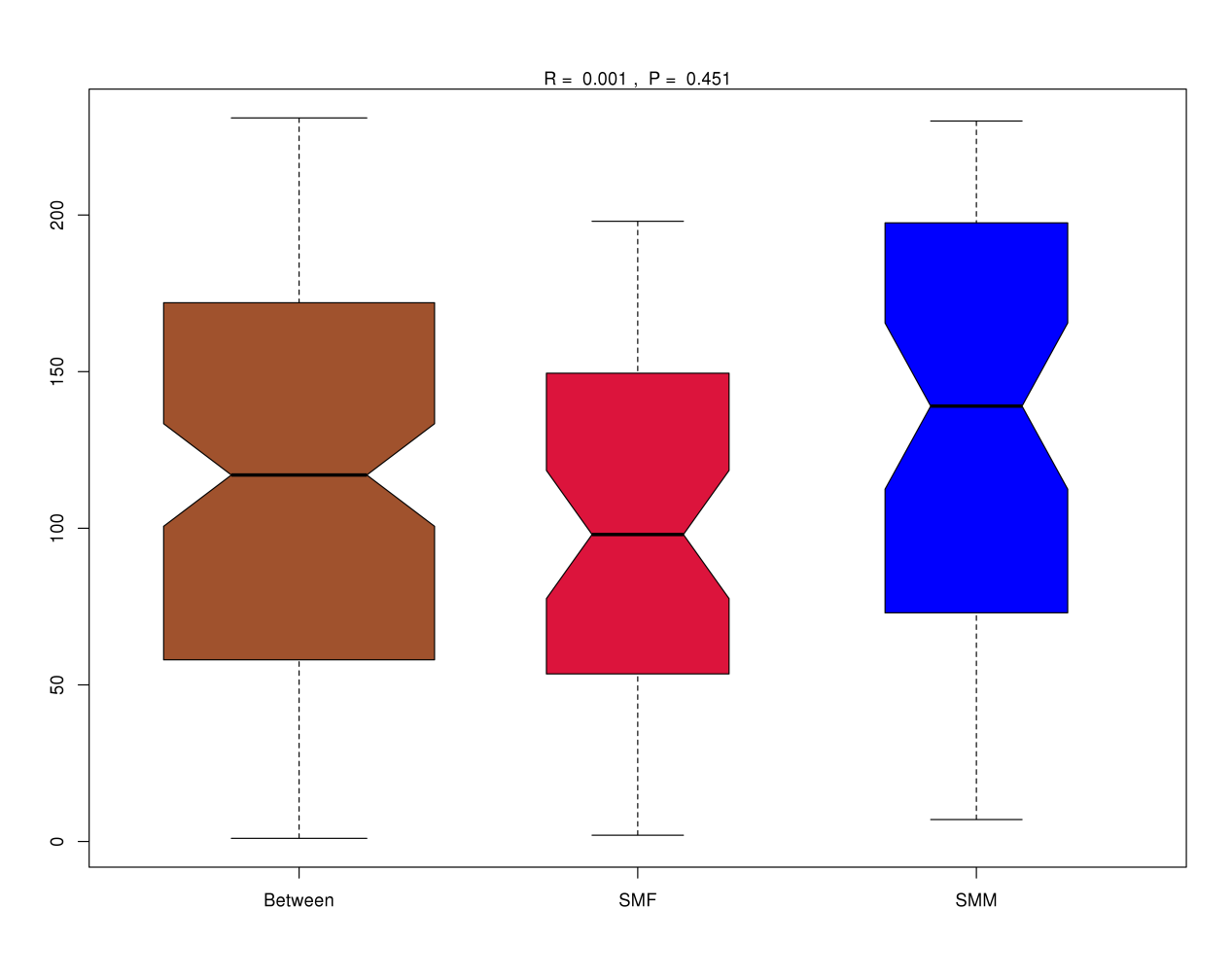

Supplement: Supplementary file 6 — Additional file 6: Fig. S6. Analysis of differences between Anosim groups of different genders. [file 13568_2021_1252_MOESM6_ESM.png]

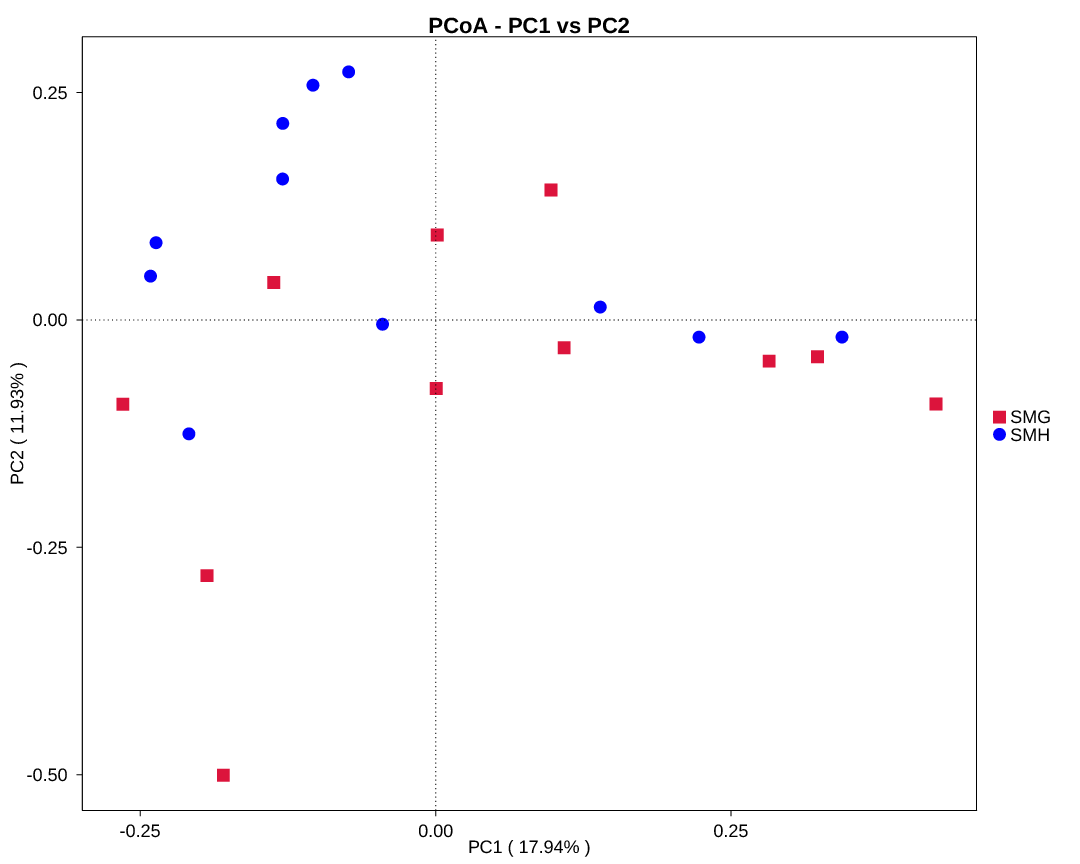

Supplement: Supplementary file 7 — Additional file 7: Fig. S7.PCoA analysis between groups of different sampling locations. [file 13568_2021_1252_MOESM7_ESM.png]

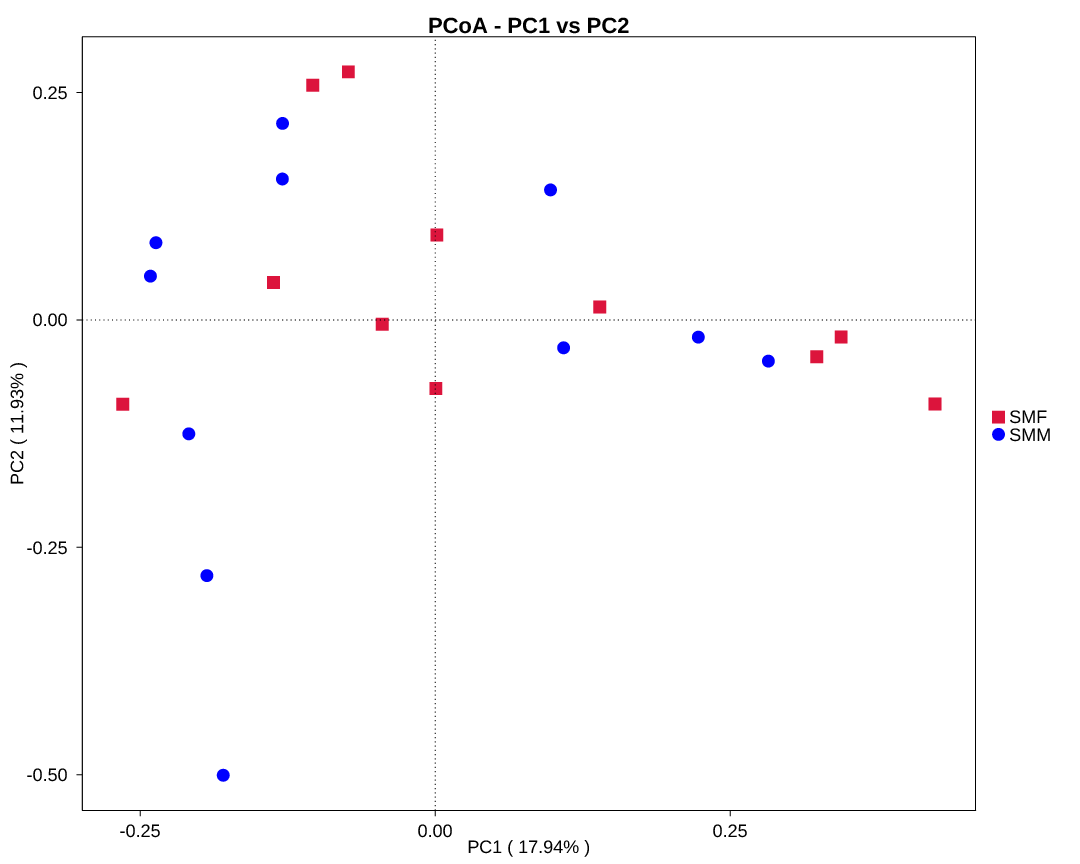

Supplement: Supplementary file 8 — Additional file 8: Fig. S8. PCoA analysis between groups of different genders. [file 13568_2021_1252_MOESM8_ESM.png]

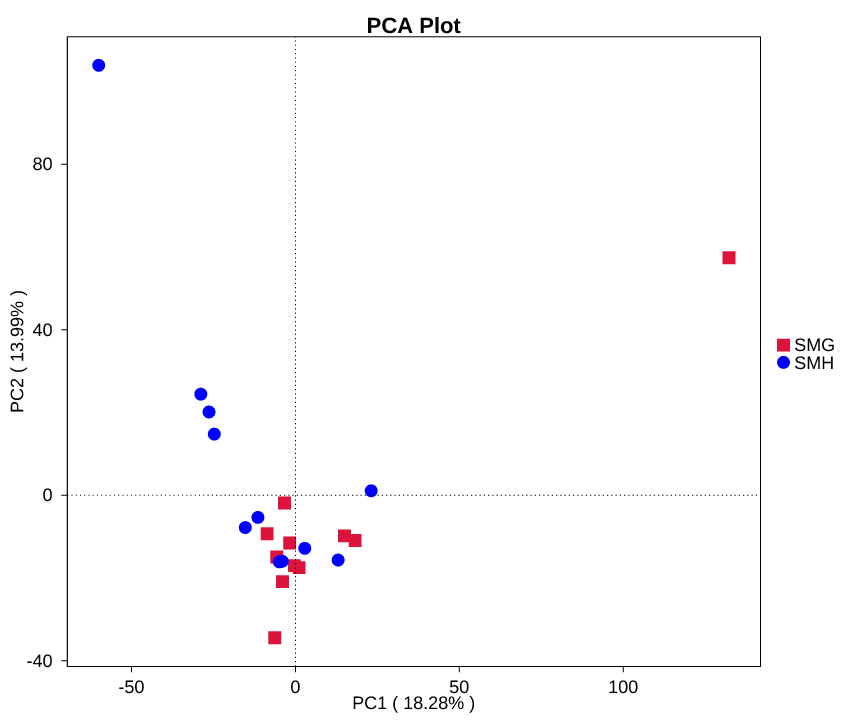

Supplement: Supplementary file 9 — Additional file 9: Fig. S9.PCA analysis between groups of different sampling locations [file 13568_2021_1252_MOESM9_ESM.png]

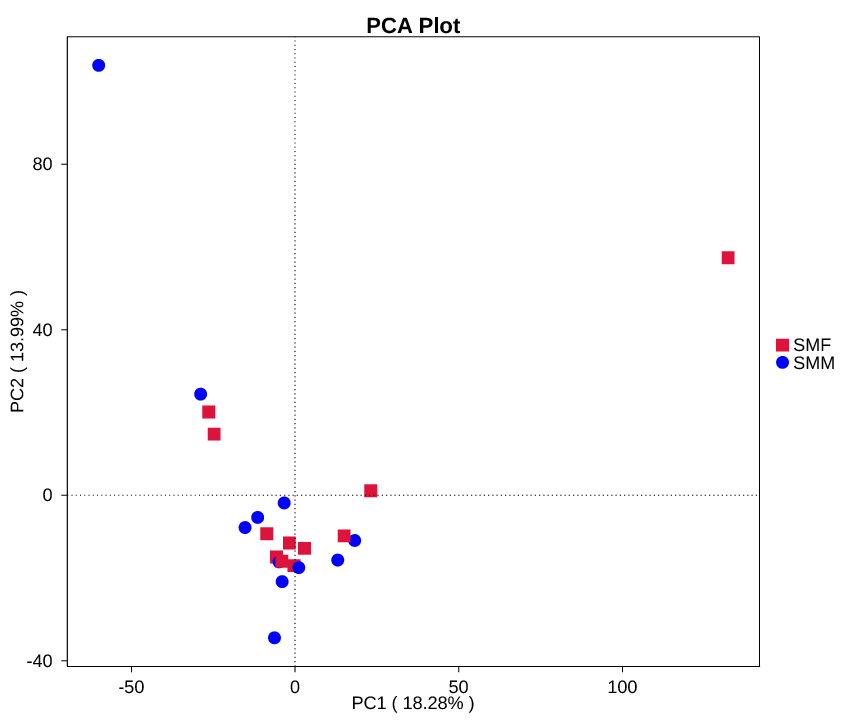

Supplement: Supplementary file 10 — Additional file 10: Fig. S10.PCA analysis between groups of different genders. [file 13568_2021_1252_MOESM10_ESM.png]

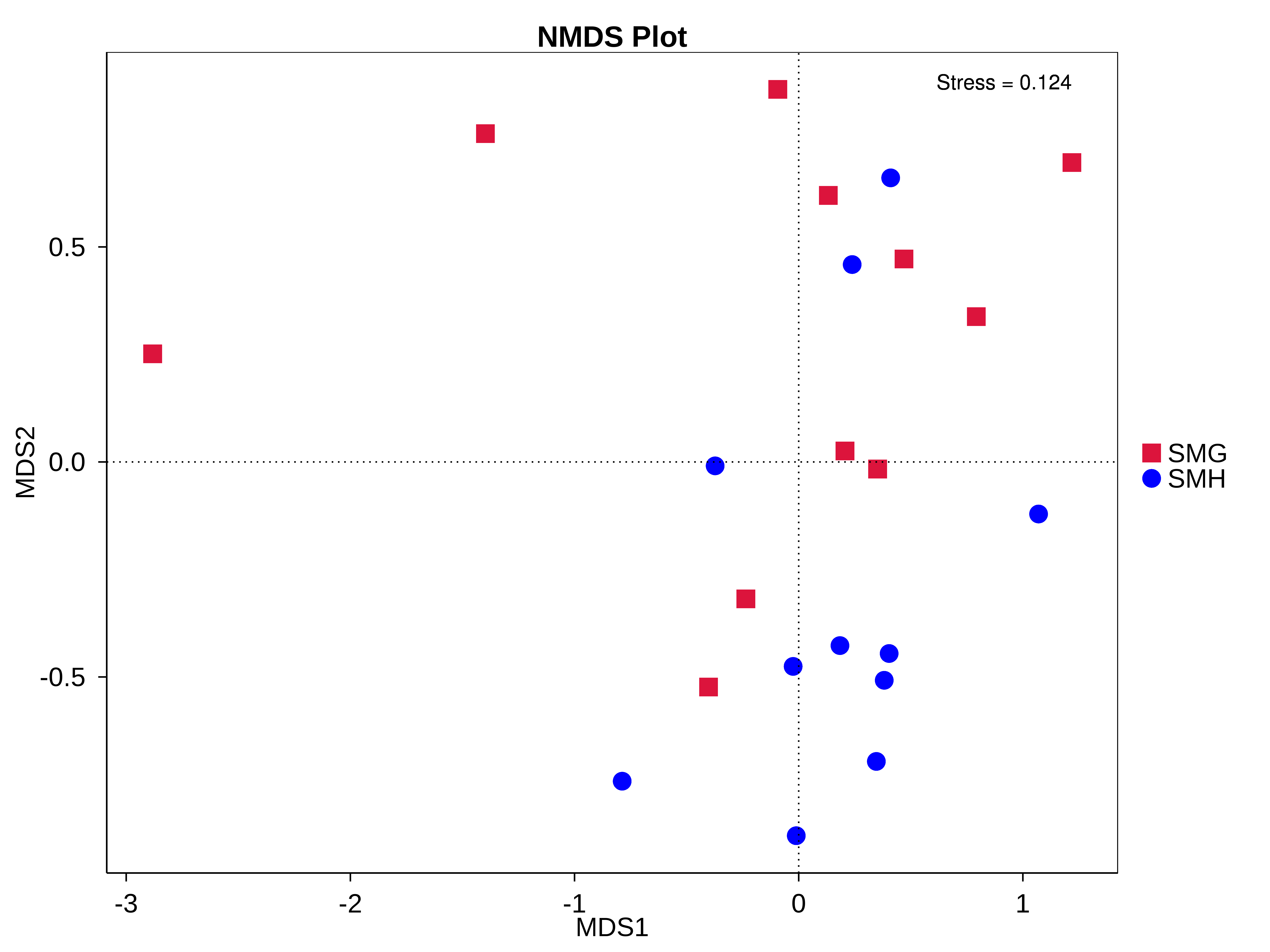

Supplement: Supplementary file 11 — Additional file 11: Fig. S11.NMDS analysis between groups of different sampling locations. [file 13568_2021_1252_MOESM11_ESM.png]

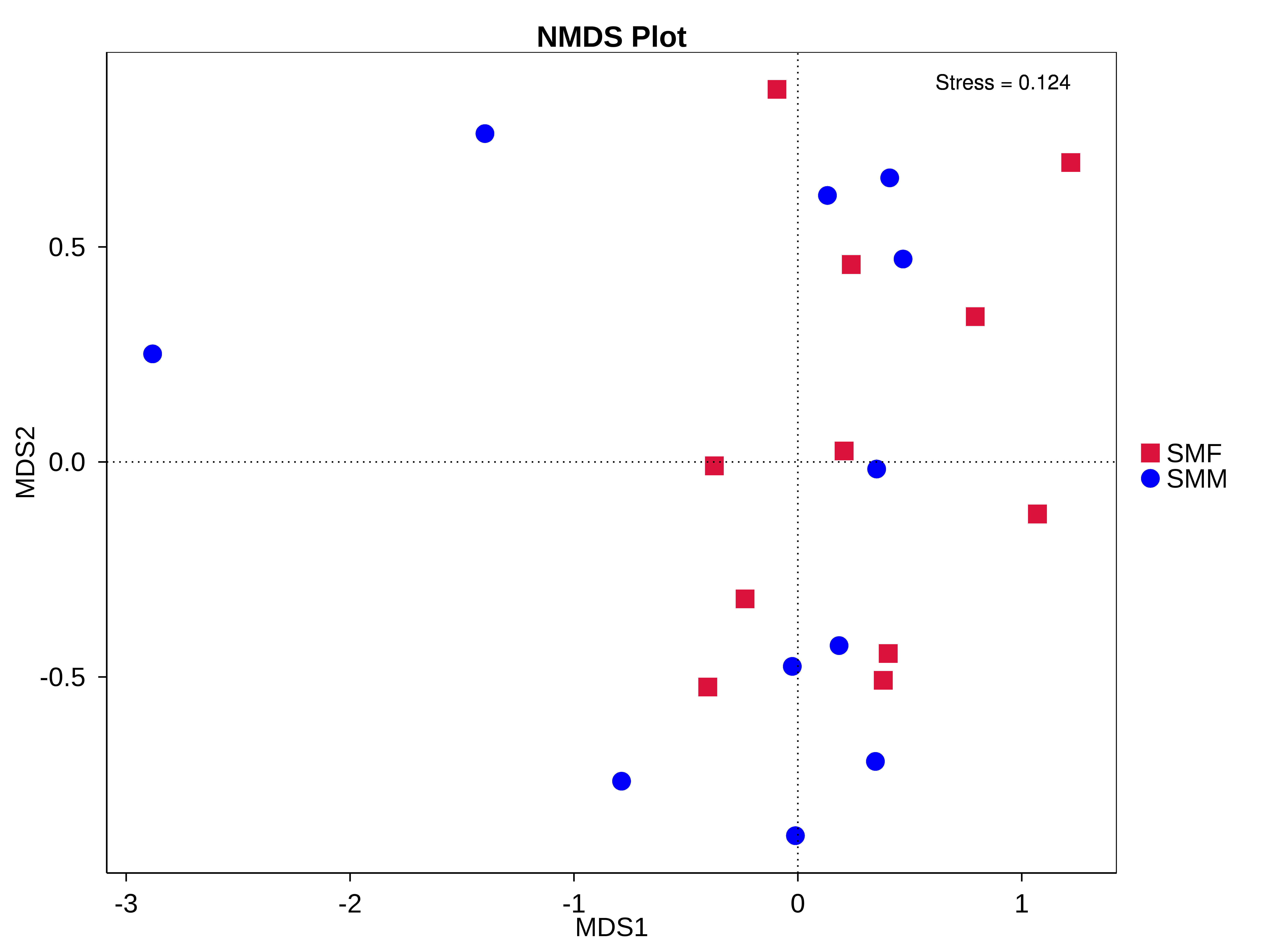

Supplement: Supplementary file 12 — Additional file 12: Fig. S12.NMDS analysis between groups of different genders. [file 13568_2021_1252_MOESM12_ESM.png]

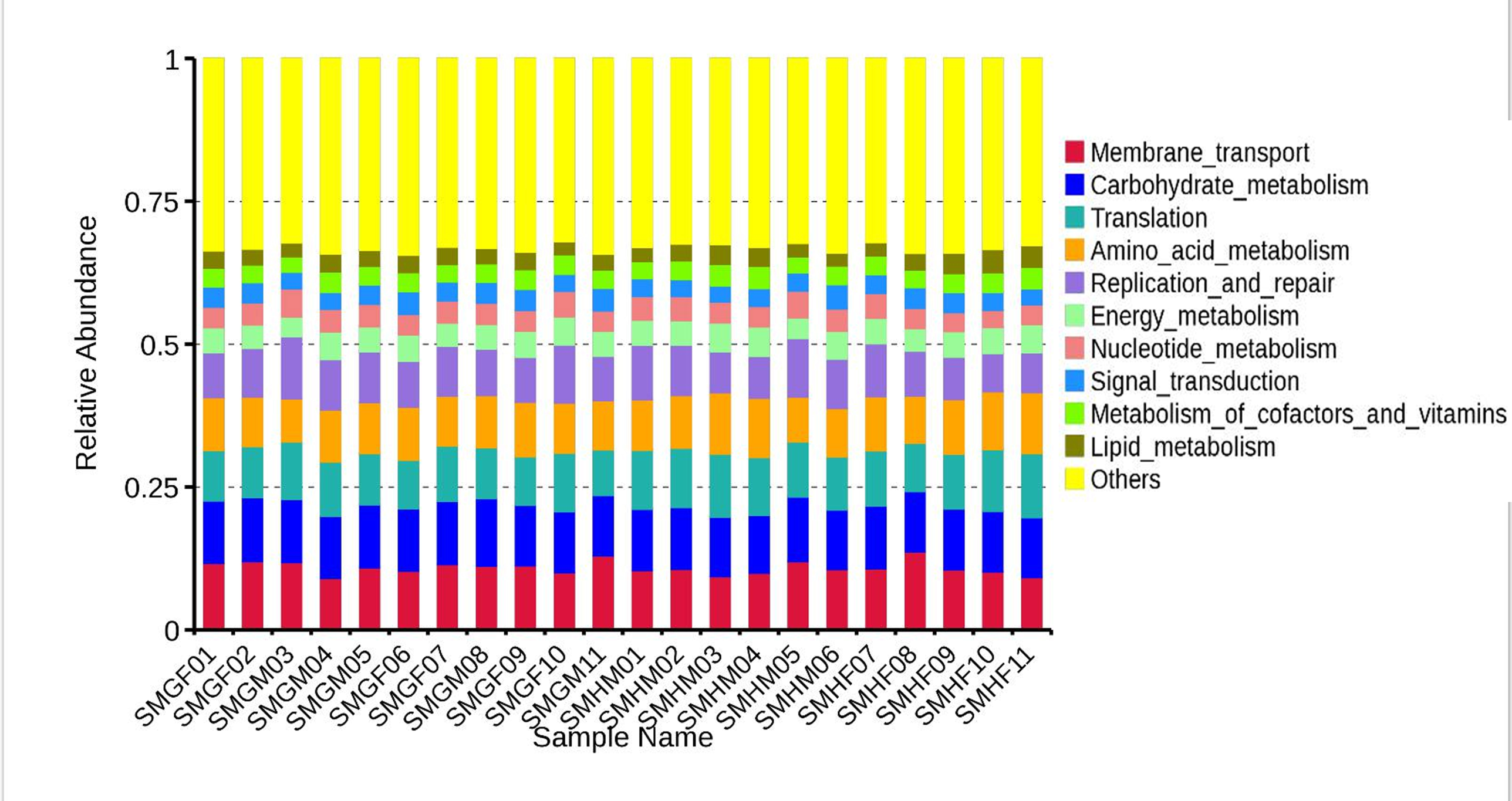

Supplement: Supplementary file 13 — Additional file 13: Fig. S13. Tax4Fun Function annotation histogram of relative abundance of level 2. [file 13568_2021_1252_MOESM13_ESM.jpg]
