# Supplementary material for: Habitat environmental factors influence intestinal microbial diversity of the short-faced moles (Scaptochirus moschata)
Source: AMB Express. 2021 Jun 23;11:93. doi: 10.1186/s13568-021-01252-2 (PMC8222469; doi:10.1186/s13568-021-01252-2)
Supplement: Supplementary file 14 — Additional file 14: Table S1. Soil physical and chemical properties in two regions (Mean±SD). [file 13568_2021_1252_MOESM14_ESM.docx]

Table S1. Soil physical and chemical properties in two regions (Mean±SD).

| Sampling Locations | Dry matter | Water | Capacity | pH | Electrical conductivity | Cation exchange capacity | Exchange sodium | Organic matter |
| --- | --- | --- | --- | --- | --- | --- | --- | --- |
|  | （%） | （%） | （g/cm3） |  | （µs/cm） | （cmol/kg） | （cmol(Na+)/kg） | （g/kg） |
| Guanxian | 94.13 | 6.29 | 1.19 | 7.81 | 134.9 | 9.91 | 0.94 | 13.3 |
| Huimin | 95.53 | 4.72 | 1.32 | 7.88 | 145.8 | 4.91 | 0.22 | 6.7 |
| P value | 0.52 | 0.52 | 0.46 | 0.58 | 0.33 | 0.0000079 | 0.00018 | 0.000097 |
